# Supplementary material for: A Novel Tissue-Free Method to Estimate Tumor-Derived Cell-Free DNA Quantity Using Tumor Methylation Patterns
Source: Cancers (Basel). 2023 Dec 23;16(1):82. doi: 10.3390/cancers16010082 (PMC10777919; doi:10.3390/cancers16010082)
Supplement: Supplementary file 1 [file cancers-16-00082-s001.zip › cancers-2714158-supplementary.pdf]

A Novel Tissue-Free Method to Estimate Tumor-Derived Cell-Free DNA Abundance Using Tumor Methylation Patterns

Collin A. Melton[1], Peter Freese[1,*], Yifan Zhou[1], Archana Shenoy[1], Siddhartha Bagaria[1,†], Christopher Chang[1], Chih-Chung Kuo[1], Eric Scott[1], Subashini Srinivasan[1], Gordon Cann[1], Manami Roychowdhury-Saha[1], Pei-Yun Chang[1], and Amoolya H. Singh[1]

Supplementary Methods

Data processing and statistical analysis—differentially methylated region calling

The overall DMR calling and annotation workflow is depicted in **Supplementary Methods Figure SM1**. To process the large amounts of DNA sequence data, we leveraged a custom "match tree" data structure consisting of a condensed representation of raw input fragment data. The analysis utilized a custom implementation using a 1D version of a Kd tree with a randomized surface-area heuristic [66]. A distinct interval was defined for every unique set of ≥5 contiguous CpGs contained within any fragment. Additionally, the counts of observed fragments for each distinct fragment methylation pattern were stored. Sample-level DMRs were called by traversing a match tree containing fragment count data for each cancer tissue WGBS sample using a sliding window of 5 CpGs. DMRs were defined for each cancer-indicative methylation pattern within a set of 5 contiguous CpGs if the following thresholds were passed: (1) non-cancer WGBS cfDNA aggregate frequency of the cancer-indicative methylation pattern was $<10^{-3}$ in a cohort of non-cancer samples, (2) the number of fragments spanning the 5 CpG set was ≥10 in the cancer tissue WGBS sample, and (3) frequency of the cancer-indicative methylation pattern was ≥0.2 in the cancer tissue WGBS sample.

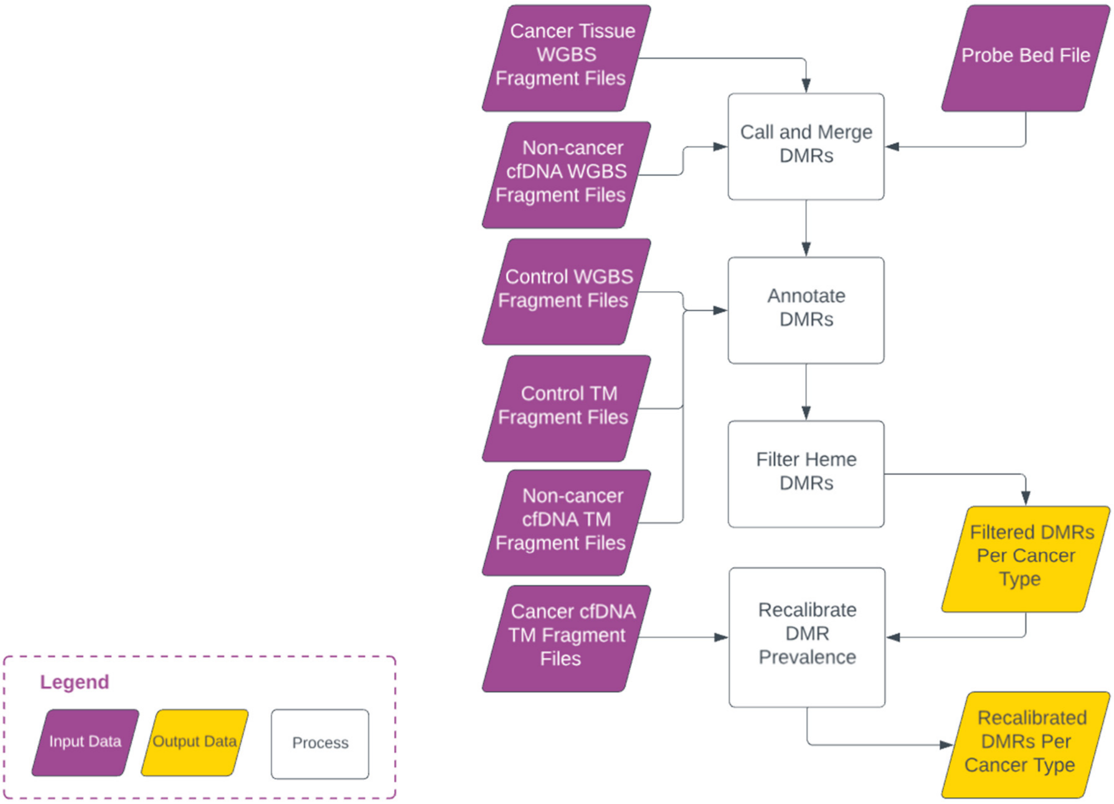

Supplementary Methods Figure SM1. DMR generation and annotation workflow. A diagram illustrating the flow of data through processes in the generation of DMR sets per cancer type. Control targeted methylation (Control TM) and control whole genome bisulfite sequencing (Control WGBS) were a contrived mixture of 100% methylated and 100% unmethylated DNA into a cell line DNA background used to estimate pull-down efficiency per DMR.

A size of ≥5 CpGs per DMR was chosen based on the criteria that the number of CpGs should be (1) large enough that the measured methylation pattern is unlikely to be observed due to bisulfite conversion failure and (2) less than or equal to the number of CpGs spanned by a typical cfDNA fragment. In a set of non-cancer targeted methylation samples, greater than 50% of fragments span 5 or more CpGs (**Supplementary Methods Figure SM2**).

(a)

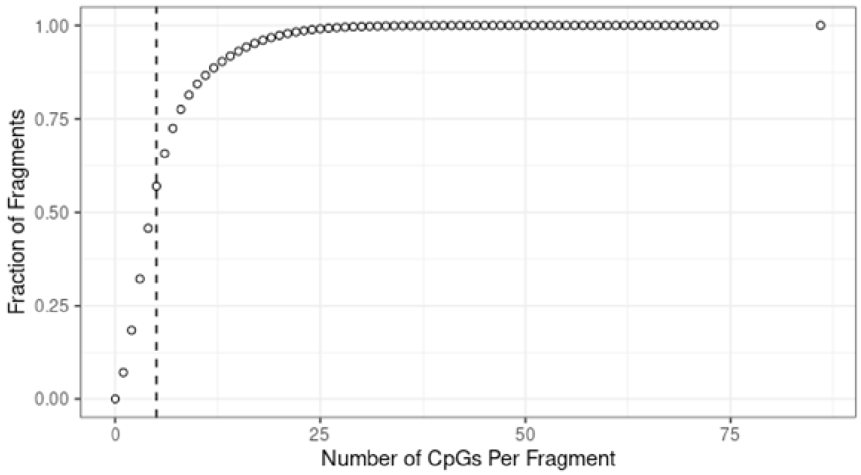

(b)

# of CpGs	Fraction of Fragments	Cumulative Fraction of Fragments
1	7.2%	7.2%
2	11.2%	18.4%
3	13.8%	32.2%
4	13.6%	45.8%
5	11.2%	57.0%
6	8.8%	65.8%
7	6.7%	72.5%
8	5.1%	77.5%
9	3.8%	81.4%
10+	18.6%	100.0%

Supplementary Methods Figure SM2. Distribution of the number of CpGs per fragment. (a) The cumulative distribution of the number of CpGs per fragment in a collection of non-cancer samples run on the GRAIL (Menlo Park, CA) targeted methylation assay. A vertical dashed line is drawn for 5 CpGs, such that 54.2% of fragments could possibly be distinguished by a 5 CpG DMR, the chosen size for a DMR in this study. (b) A table of the data plotted in (a).

The non-cancer WGBS cfDNA frequency threshold was set to be permissive so as not to filter out potentially useful methylation patterns. The fragment depth and tissue frequency thresholds were set with the intention of reducing the identification of

methylation patterns present in non-cancer cellular impurities within the tissue samples. The filters used required the observation of at least 2 unique fragments with the methylation pattern of interest and required that the frequency of this pattern be consistent with pathology tissue purity estimates that typically exceed 50%.

DMRs called in individual tissue samples were merged according to each sample's cancer label, using reported cancer labels as defined in Klein et al. 2021 [37]. Letting V_i be the set of DMRs in sample i, we defined the set of DMRs for cancer label k, V_k , as the union across all samples with label k such that $V_k = V_1 \cup V_2 \cup V_3\ldots$ After DMR merging, match trees for individual tissue samples were traversed again to compute the mean DMR frequency for all samples with at least one fragment containing the DMR.

Data processing and statistical analysis - DMR prevalence estimation calculation

To generate a mean close to the observed prevalence in the tissue set, the beta prior on the DMR prevalence was used with alpha equal to (number of tissue samples observed to have the DMR's cancer-indicative methylation pattern) x (scaling factor) + 1, and beta set to (number of tissue samples observed to not have the DMR's cancer-indicative methylation pattern) x (scaling factor) + 1. The scaling factor is included to be able to tune the prior, such that a lower scaling factor weakens the prior, and a higher scaling factor strengthens it. The prevalence for DMR i (R_i) was computed as follows:

$P(R_i \mid TF_1 \ldots TF_n, x_{i,1}, \ldots x_{i,n}) \sim P(TF_1 \ldots TF_n, x_{i,1}, \ldots x_{i,n} \mid R_i) * P(R_i)$

$P(TF_1 \ldots TF_n, x_{i,1}, \ldots x_{i,n} \mid R_i)$ is computed as $\Pi P(TF_j, x_{i,j} \mid R_i)$

$P(TF_j, x_{i,j} \mid R_i)$ is computed as described below, and $x_{i,j}$ is the count of fragments in the jth sample (of n total samples) containing the ith variant. TFj is the tumor fraction for the jth sample, defined as the proportion of cfDNA derived from the tumor.

Data processing and statistical analysis—tumor methylated fraction estimation calculations

TF was inferred from the count of cancer-indicative methylation pattern fragments x_j at each DMR j in a set of m DMRs as follows:

$P(TF \mid x_j \ldots x_m) \sim P(x_i \ldots x_n \mid TF) * P(TF)$

where $P(TF) \sim Beta(1, 1)$.

The likelihood of observing a vector of fragment counts across a set of DMRs was modeled as a 2-component mixture of fragments derived from shedding cancer cells and fragments derived from a background noise process. Fragment counts at each DMR were assumed to be independent.

$P(x_i \ldots x_n \mid TF) = \Pi P(x_i \mid TF)$

The per-DMR likelihood for each DMR count was computed as the prevalence-weighted Poisson likelihood of the observed fragment.

$\Pi P(x_i \mid TF) = R_i * Pois(x_i \mid \text{DMR in shedding tumor}) +$
$(1 - R_i) * Pois(x_i \mid \text{DMR is not in the shedding tumor})$.

The fragment count at each DMR site given the DMR is present in the shedding tumor was modeled as a Poisson distribution with parameter lambda calculated as a mixture of tumor-derived and non-tumor-derived cfDNA.

λ = [TF * (tissue DMR cancer indicative methylation pattern frequency) + (1 - TF) * (noise rate)] * (estimated depth).

Tissue cancer-indicative methylation pattern frequency was defined as the aggregate cancer-indicative methylation pattern frequency (i.e., the number of fragments with the DMR's cancer-indicative methylation pattern out of the total number of fragments) across all cancer tissue WGBS samples with 1 or more fragments containing the DMR cancer-indicative methylation pattern. Depth was approximated as (empirical pull-down efficiency for fully methylated or fully unmethylated control DNA) * (estimated fragment count in the pre pull-down library). The noise rate was estimated as the mean from an aggregated set of non-cancer targeted methylation and non-cancer WGBS samples. Prior to TF estimation, DMRs were filtered to include those that pass the following filters:

1. Not in the top 5% of raw sample counts;
2. On an autosome;
3. Completely methylated or completely unmethylated, as these variants have better pull-down bias estimates;

4. Empirically determined to have probes that pull down the intended abnormally methylated DNA;
5. Have an estimated noise rate below 1/10,000.

To convert the TF estimate to an allele fraction estimate, the posterior TF values were multiplied by a robust estimate of the central tendency of the tissue cancer-indicative methylation pattern frequency distribution. The measure used here was one-half of the 95th percentile of all DMRs with greater than 50% prevalence. This measure can be thought of as estimating the tumor purity in the reference DMR dataset by using the 95th percentile of the tissue frequency distribution and dividing by 2 to estimate the contribution of a heterozygous allele. The modeled TF measurement assumes 100% tumor purity as we use the raw observed tissue DMR cancer-indicative methylation pattern frequency in computing the Poisson lambda in the likelihood calculation for each DMR. In converting TF to an estimate of allele fraction, we post hoc account for the tumor purity in the reference dataset by using the 95th quantile of the tissue frequency distribution and furthermore scale by a factor of one-half to calibrate TMeF to be more comparable to small variant based allele fraction estimates.

Synthetic dilution calculations

Synthetic dilution series were generated via binomial sampling of fragment count data for each DMR across a series of mixing fractions for pairs of cancer and non-cancer samples. The mixing fractions were post hoc corrected for the difference in coverage between the undiluted cancer (C_C) and non-cancer (C_{NC}) samples used in each dilution series such that the corrected mixing fraction $rc = \frac{r \cdot C_C}{r \cdot C_C + (1-r) \cdot C_{NC}}$. If the allele fraction for the cancer sample is AF_C and the corrected mixing fraction is rc, the theoretical allele fraction of the titrated sample would be $rc \cdot AF_C$.

For each DMR in the cancer sample, the matching DMR in the non-cancer sample was identified based on position and methylation pattern. For each DMR, given the mixing fraction (r), the number of fragments containing each DMR in the cancer and non-cancer samples, respectively (m_C, m_{NC}), and the number of fragments not containing each DMR in the cancer and non-cancer samples, respectively (u_C, u_{NC}), the titrated fragment counts with and without the DMR, respectively (m_T, u_T), were

computed using binomial sampling such that $m_T \sim Bin(m_C, r) + Bin(m_{NC}, 1-r)$ and $u_T \sim Bin(u_C, r) + Bin(u_{NC}, 1-r)$.

Biophysical modeling of ctDNA shedding

Linear modeling was performed to fit regression models of the form log(TMeF) ~ log(tumor size). This corresponds to a model of TMeF ~ slope * (tumor size)$^{\text{scaling factor}}$. For a true biological link between TMeF and tumor size, a positive and significant slope and a positive ≤3 scaling factor would be expected. In biophysical terms, a scaling factor of 2 is consistent with DNA shed proportional to tumor surface area and a scaling factor of 3 is consistent with DNA shed proportional to total tumor volume. Important caveats of this overall approach include: (1) that a single maximum tumor size may not adequately reflect total tumor size if multiple lesions are present; (2) TMeF values taper off around ~10^{-5} so modeling in low-shedding tumors could underestimate the slope and scaling factor if a substantial number of true cTAF values are <10^{-5}; and (3) the model simplifies the complex biological underpinnings of ctDNA shedding, excluding important factors such as mitotic rate estimates, which were unavailable for the analyzed sample set.
