# Supplementary material for: A Novel Tissue-Free Method to Estimate Tumor-Derived Cell-Free DNA Quantity Using Tumor Methylation Patterns
Source: Cancers (Basel). 2023 Dec 23;16(1):82. doi: 10.3390/cancers16010082 (PMC10777919; doi:10.3390/cancers16010082)
Supplement: Supplementary file 1 [file cancers-16-00082-s001.zip › Melton.Cancers.TMeF_Supplementary figures_no change.pdf]

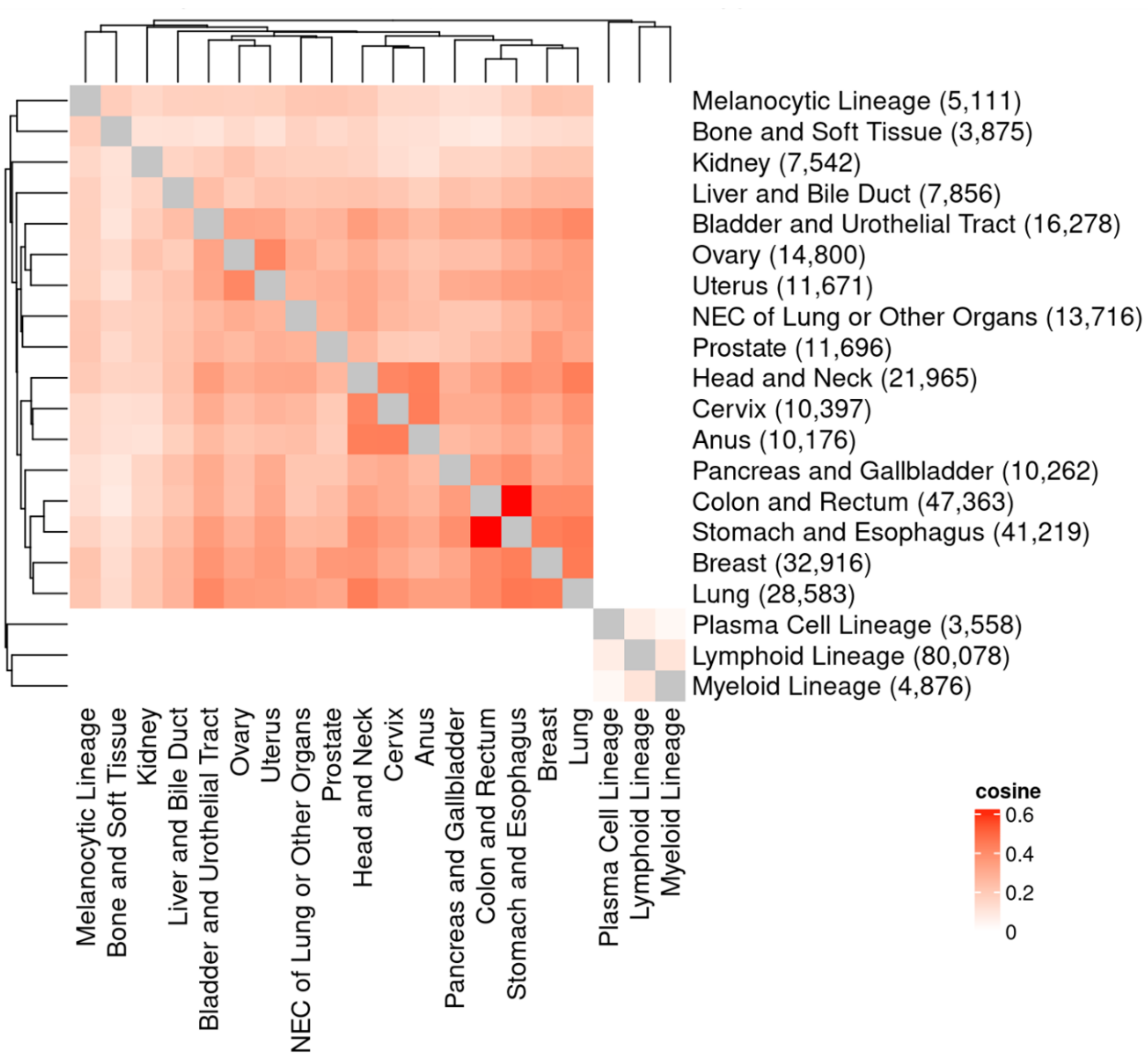

Figure S1. DMR clustering across cancer labels. A heatmap depicting the cosine similarity between DMRs defined for each cancer label relative to non-cancer cfDNA.

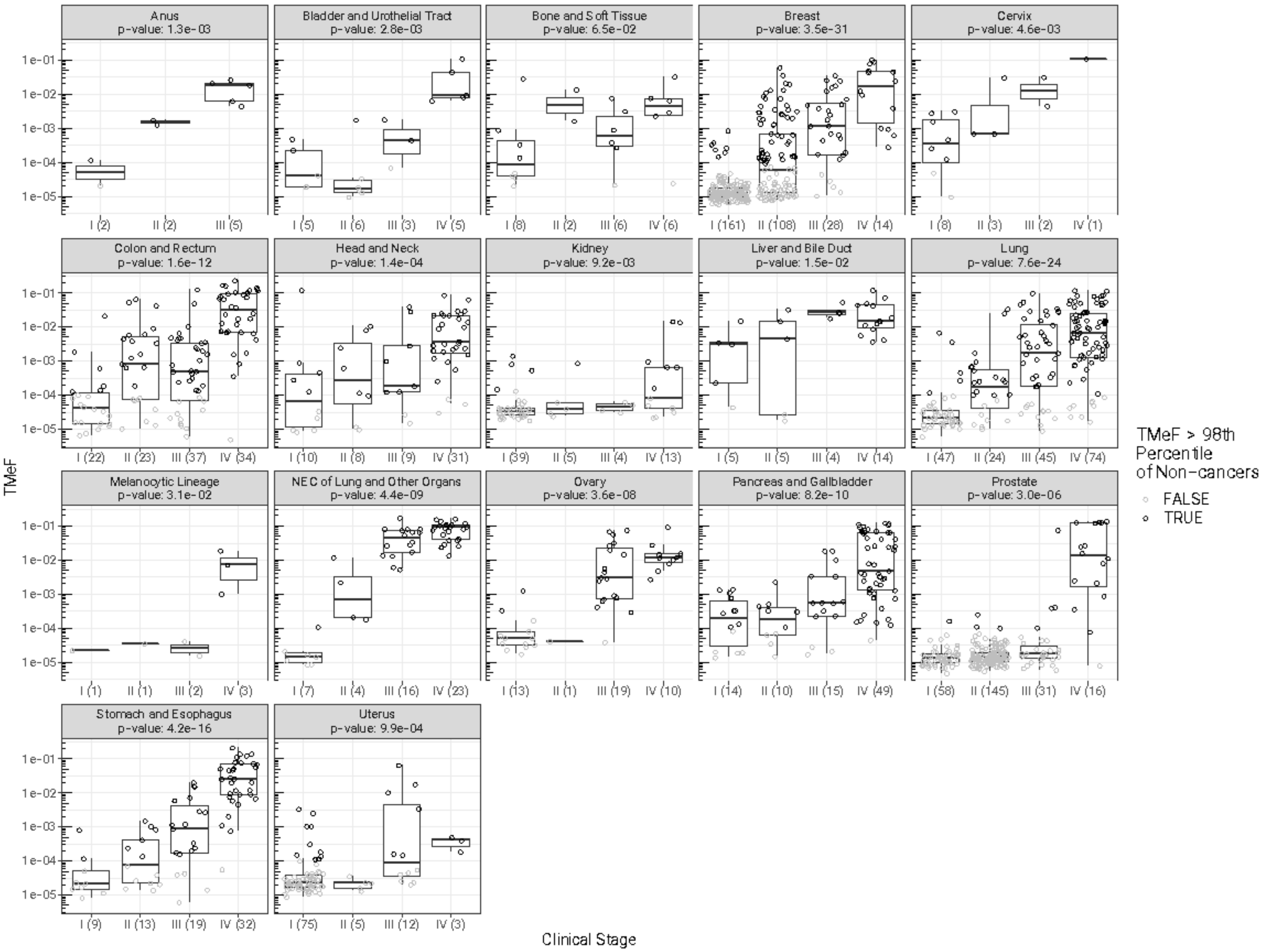

Figure S2. TMeF correlation with clinical stage by cancer label. TMeF for 1434 pre-treatment, solid cancer samples from CCGA substudy 3 was plotted by clinical stage and faceted by cancer label. Points were colored gray if the sample's TMeF was lower than the 98th percentile of TMeFs computed on a set of non-cancer samples to indicate that these TMeF values were less reliable. Statistical significance was assessed using a Spearman rank correlation of TMeF versus stage and corrected for multiple testing using a Benjamini Hochberg FDR correction.

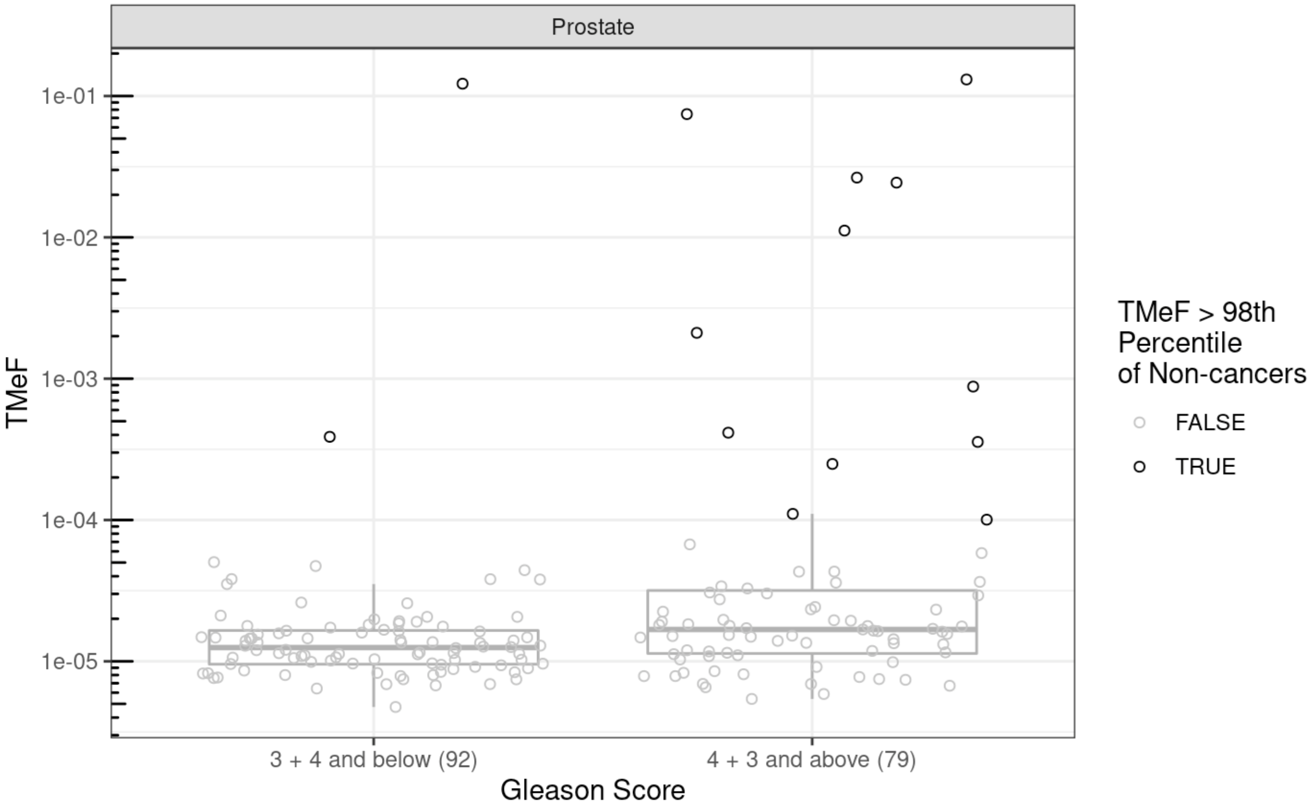

Figure S3. TMeF correlation with Gleason score in prostate cancer. TMeF for 171 prostate cancer samples was plotted by Gleason score category, 3 + 4 and below or 4 + 3 and above. Points were colored gray if the sample's TMeF was lower than the 98th percentile of TMeFs computed on a set of non-cancer samples to indicate that these TMeF values were less accurate. A p-value of 0.0035 was determined by dichotomizing each Gleason score group into samples with TMeF greater than and less than the 98th percentile of non-cancer TMeFs, then performing Fisher's exact test for count data.

(a)

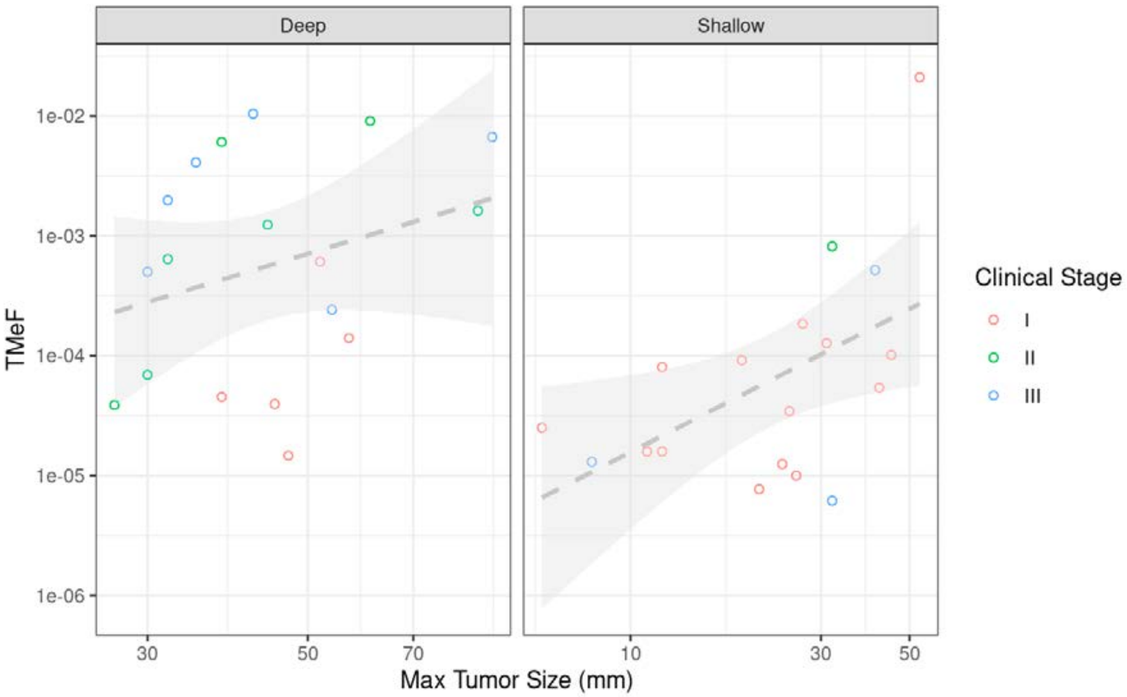

(b)

Term	Value	P-value	Invasion
Slope	10^{-14}	0.009*	Deep
Tumor-size scaling factor	1.8	0.2	Deep
Slope	10^{-15}	<0.001	Shallow
Tumor-size scaling factor	1.7	0.031*	Shallow

Figure S4. Association of TMeF with tumor size in colorectal cancer. (a) TMeF was plotted against the maximum tumor size for deep and shallow invading colorectal cancers as previously modeled in Bredno *et al*.[1] (b) Tumor shedding as a function of tumor size was modeled (see Methods) to ascertain both a measurement of the linear slope between TMeF and tumor size (observed as an intercept offset on the log-log plot) and the scaling factor associated with tumor size (observed as a slope on the log-log plot). *p<0.05.

[1]Bredno J, Lipson J, Venn O, Aravanis AM, Jamshidi A. Clinical correlates of circulating cell-free DNA tumor fraction. Baucknecht M, ed. PLOS ONE. 2021;16(8):e0256436. doi:10.1371/journal.pone.0256436

(a)

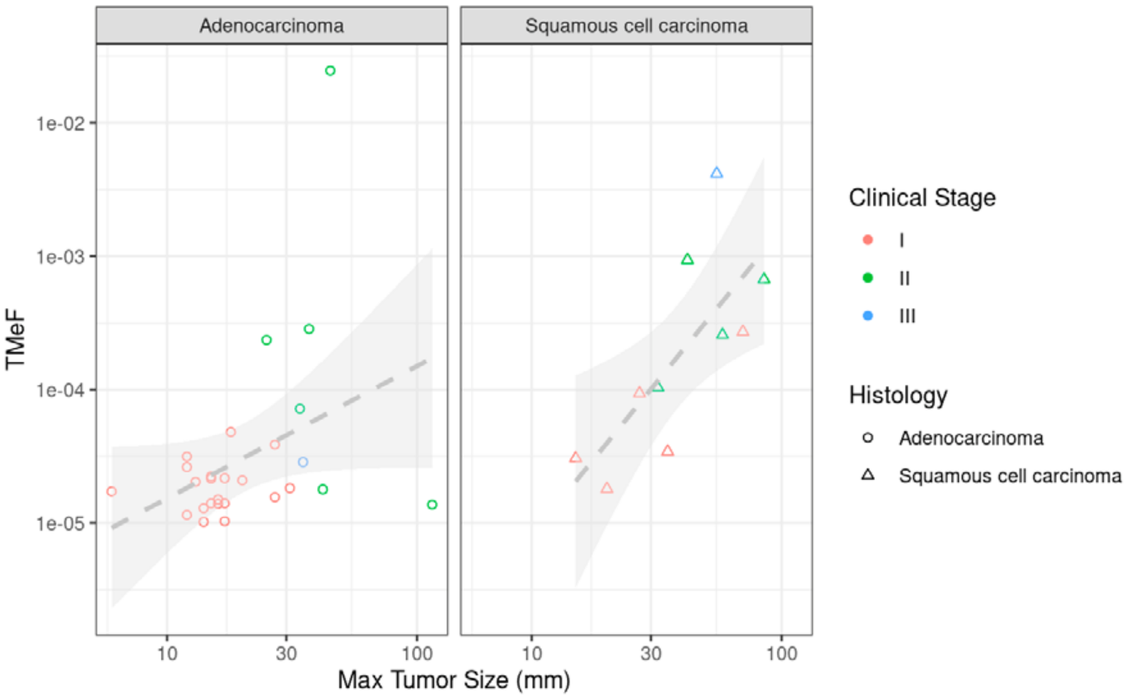

(b)

Term	Value	P-value	Histology
Slope	10^{-13}	<0.001*	Adeno
Tumor-size scaling factor	1.0	0.048*	Adeno
Slope	10^{-17}	<0.001*	SCC
Tumor-size scaling factor	2.3	0.012*	SCC

Figure S5. Association of TMeF with tumor size in NSCLC. (a) TMeF was plotted against the maximum tumor size for lung adenocarcinoma and lung squamous cell carcinoma. (b) Tumor shedding as a function of tumor size was modeled (see Methods) to ascertain both a measurement of the linear slope between TMeF and tumor size (observed as an intercept offset on the log-log plot) and the scaling factor associated with tumor size (observed as a slope on the log-log plot). *p<0.05.

(a)

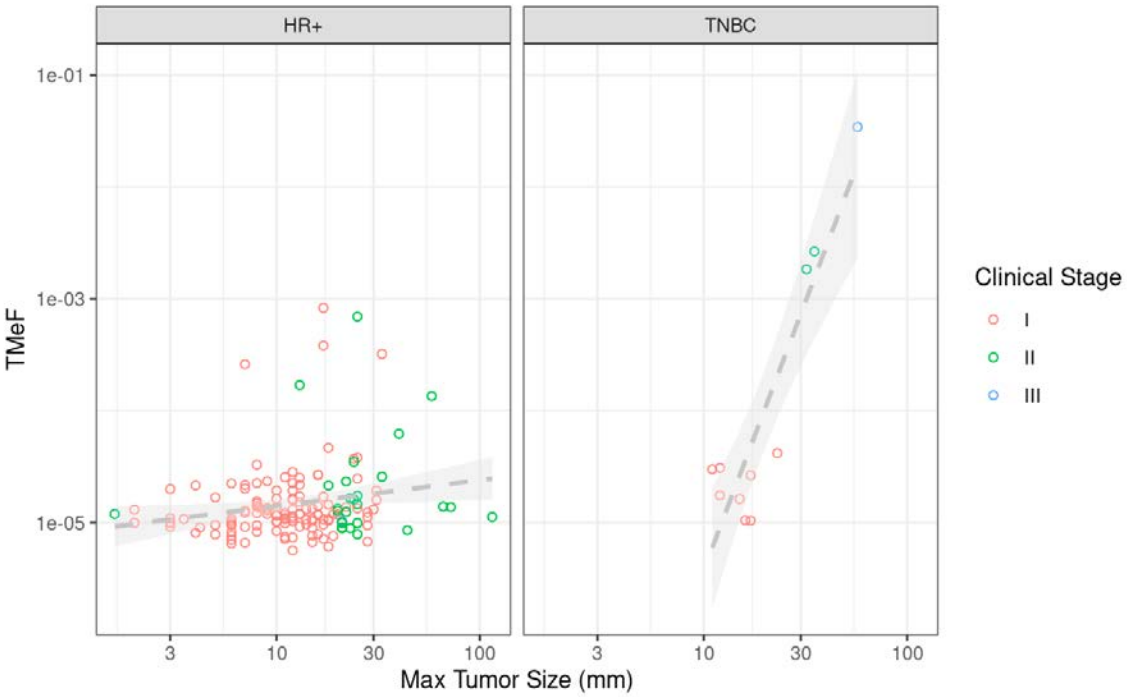

(b)

Term	Value	P-value	Subtype
Slope	10^{-12}	<0.001*	HR+
Tumor-size scaling factor	0.23	0.02*	HR+
Slope	10^{-24}	<0.001*	TNBC
Tumor-size scaling factor	4.8	<0.001*	TNBC

Figure S6. Association of TMeF with tumor size in breast cancer. (a) TMeF was plotted against the maximum tumor size for hormone receptor positive (HR+) and triple negative breast cancer (TNBC). (b) Tumor shedding as a function of tumor size was modeled (see Methods) to ascertain both a measurement of the linear slope between TMeF and tumor size (observed as an intercept offset on the log-log plot) and the scaling factor associated with tumor size (observed as a slope on the log-log plot). The HR+ model is included for completeness; however, the fit was poor. This may be due to the limit of quantification of TMeF and/or the importance of unmodeled tumor characteristics. *p<0.05.

(a)

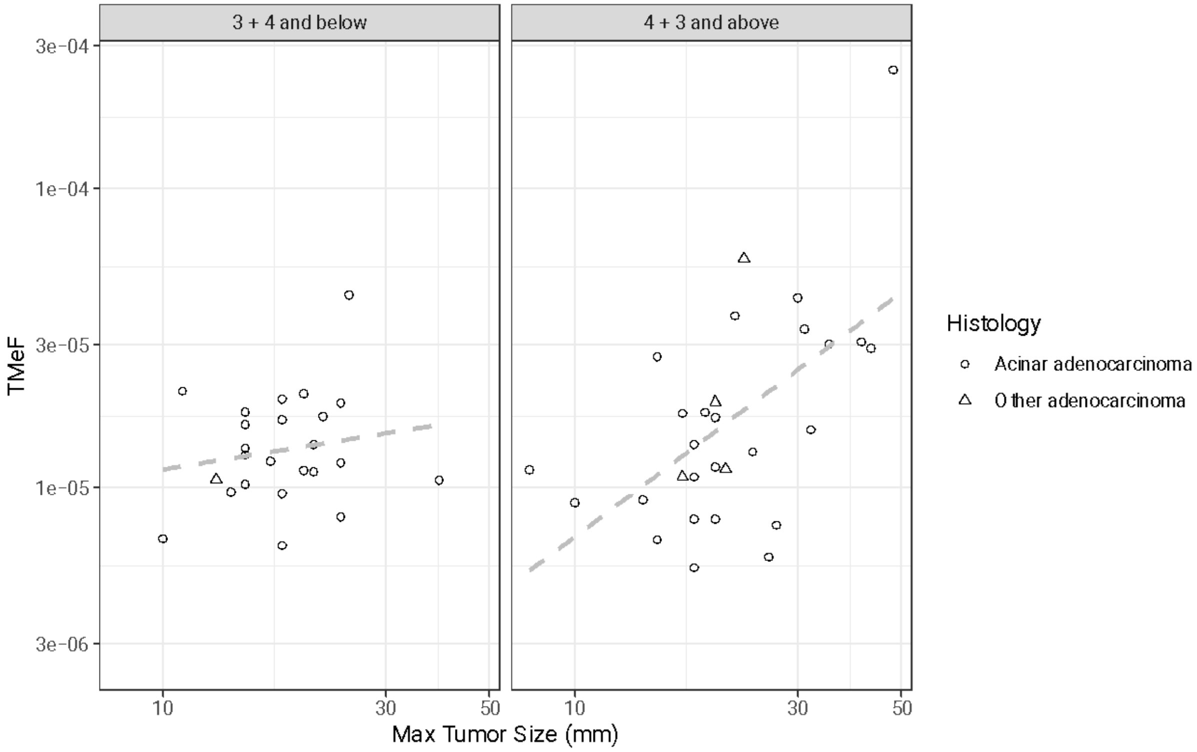

(b)

Term	Value	P-value	Gleason
Slope w/ lymph node involvement	10^{-11}	<0.001*	3 + 4 and below
Slope w/o lymph node involvement	10^{-12}	<0.001*	3 + 4 and below
Tumor-size scaling factor	0.25	0.5	3 + 4 and below
Slope w/ lymph node involvement	10^{-13}	<0.001*	4 + 3 and above
Slope w/o lymph node involvement	10^{-14}	<0.001*	4 + 3 and above
Tumor-size scaling factor	1	<0.001*	4 + 3 and above

Figure S7. Association of TMeF with tumor size in prostate cancer. (a) TMeF was plotted against the maximum tumor size for Gleason 3 + 4 and below and Gleason 4 + 3 and above prostate cancer participants. Involvement of one or more lymph nodes was modeled as a factor altering the slope. (b) Tumor shedding as a function of tumor size was modeled (see Methods) to ascertain both a measurement of the linear slope between TMeF and tumor size (observed as an intercept offset on the log-log plot) and the scaling factor associated with tumor size (observed as a slope on the log-log plot). The models are included for completeness; however, the fits were poor. This may be due to the limit of quantification of TMeF and/or the importance of unmodeled tumor characteristics. *p<0.05.

(a)

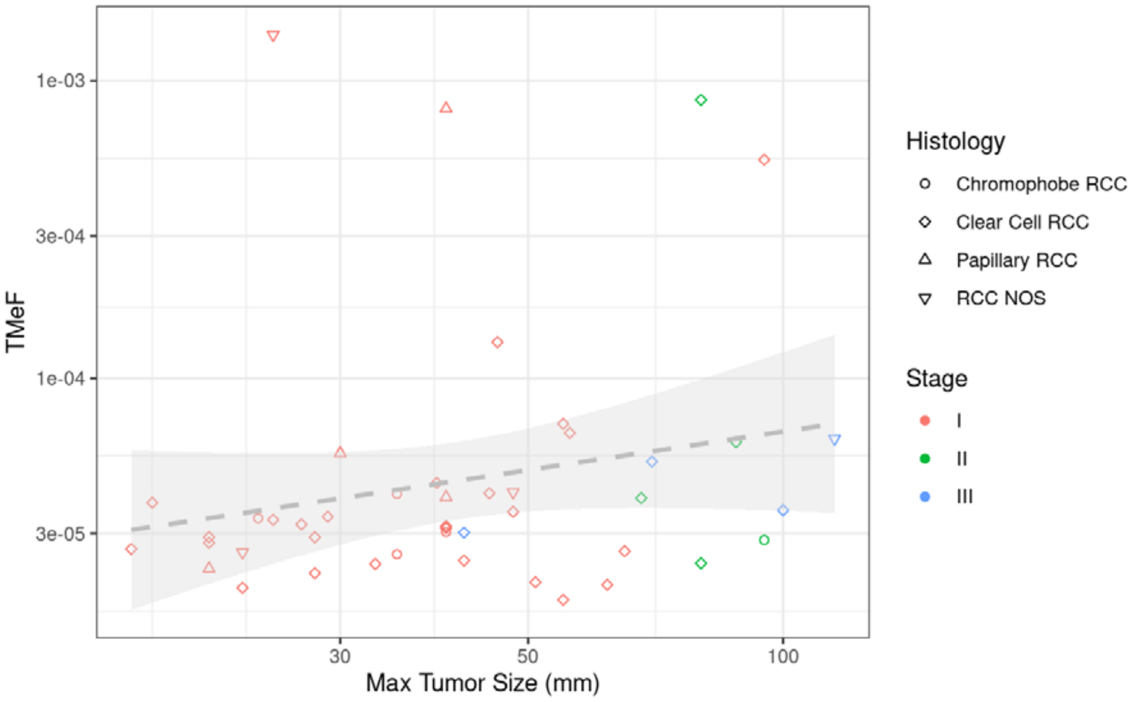

(b)

Term	Value	P-value
Slope	10^{-12}	<0.001*
Tumor-size scaling factor	0.43	0.2

Figure S8. Association of TMeF with tumor size in kidney cancer. (a) TMeF was plotted against the maximum tumor size for kidney cancers. (b) Tumor shedding as a function of tumor size was modeled (see Methods) to ascertain both a measurement of the linear slope between TMeF and tumor size (observed as an intercept offset on the log-log plot) and the scaling factor associated with tumor size (observed as a slope on the log-log plot). The model is included for completeness; however, the fit was poor. This may be due to the limit of quantification of TMeF and/or the importance of unmodeled tumor characteristics. *$p<0.05$.

(a)

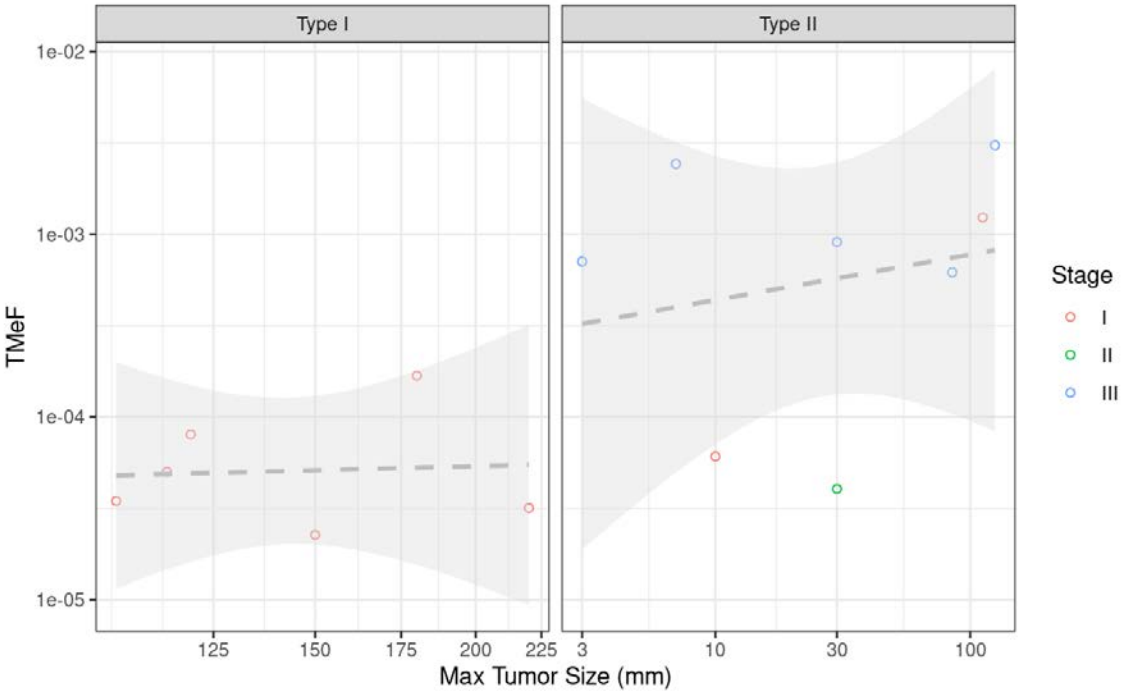

(b)

Term	Value	P-value	Subtype
Slope	10^{-11}	0.3	Type I
Tumor-size scaling factor	0.18	>0.9	Type I
Slope	$10^{-8.3}$	<0.001*	Type II
Tumor-size scaling factor	0.25	0.5	Type II

Figure S9. Association of TMeF with tumor size in ovarian cancer. (a) TMeF was plotted against the maximum tumor size for type I and type II ovarian cancer participants. (b) Tumor shedding as a function of tumor size was modeled (see Methods) to ascertain both a measurement of the linear slope between TMeF and tumor size (observed as an intercept offset on the log-log plot) and the scaling factor associated with tumor size (observed as a slope on the log-log plot). *p<0.05.

(a)

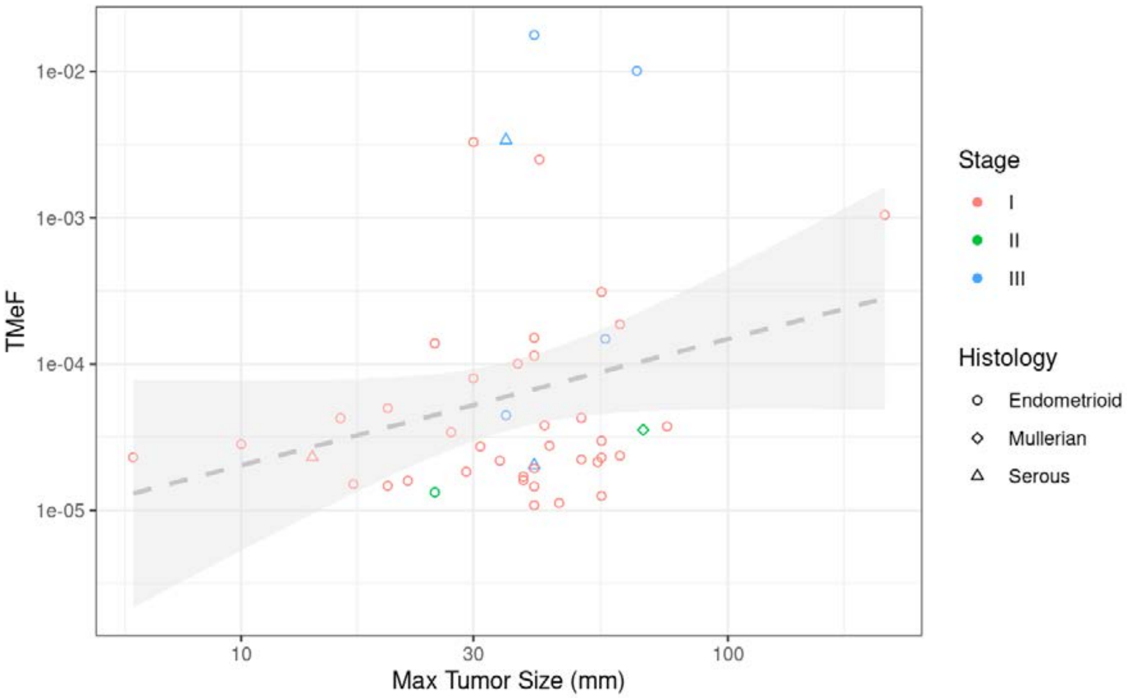

(b)

Term	Value	P-value
Slope	10^{-13}	<0.001*
Tumor-size scaling factor	0.84	0.07

Figure S10. Association of TMeF with tumor size in uterine cancer. (a) TMeF was plotted against the maximum tumor size for uterine cancer participants. (b) Tumor shedding as a function of tumor size was modeled (see Methods) to ascertain both a measurement of the linear slope between TMeF and tumor size (observed as an intercept offset on the log-log plot) and the scaling factor associated with tumor size (observed as a slope on the log-log plot). The model is included for completeness; however, the fit was poor. This may be due to the limit of quantification of TMeF and/or the importance of unmodeled tumor characteristics. *p<0.05.

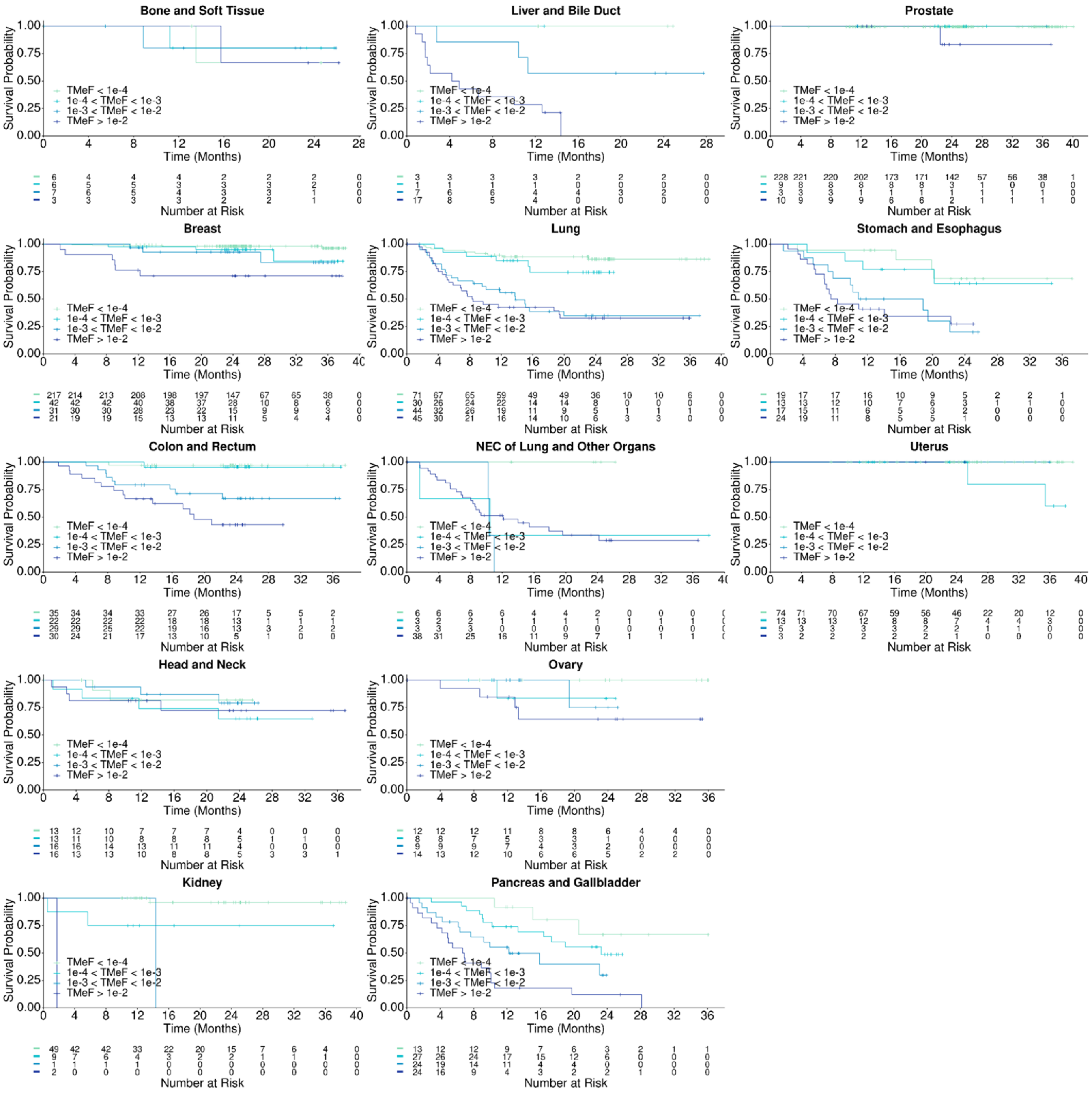

Figure S11. Survival stratified by TMeF for individual cancer labels.
Participant samples were stratified by their TMeF, and Kaplan Meier plots were generated for cancer labels where 20 or more participants had survival data available.

(a)

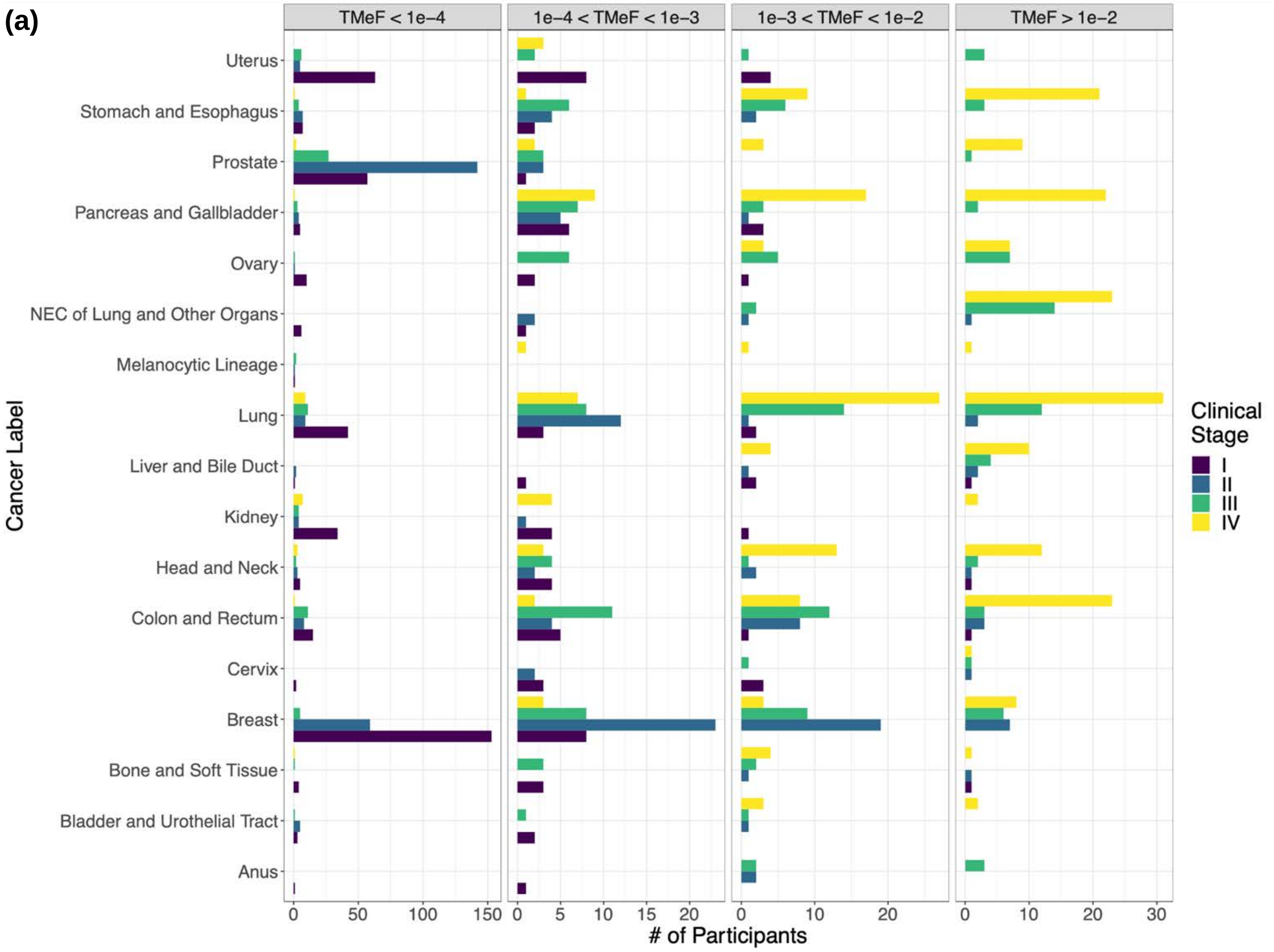

(b)

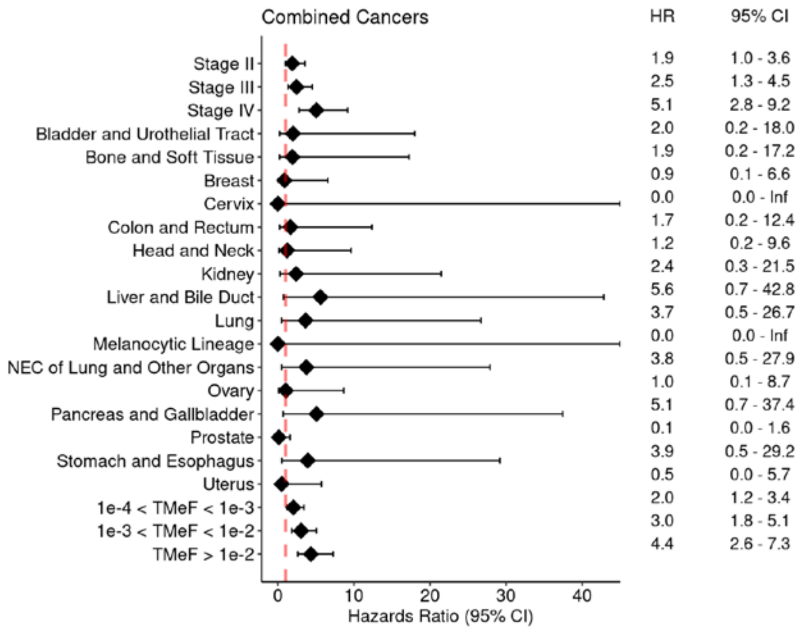

Figure S12. Cancer spectrum by clinical stage and TMeF stratum.
(a) TMeF was calculated for 1434 pre-treatment, solid cancer samples from CCGA substudy 3. Counts of samples stratified by clinical stage and cancer label are shown for each of 4 TMeF stratum. Low TMeF samples were enriched for earlier-stage cancers; whereas, high TMeF samples were enriched for later-stages. (b) A Cox proportional hazards model with clinical stage, cancer label, and stratified TMeF was fit across all cancer samples. A forest plot depicts the hazard ratios for each covariate. Caveats of the model and associated hazard ratios are described in the Results.

Reported Lower Limit of Accurate Tissue-Free Quantification

Assay Type	<0.1%	0.1-1%	1–10%	Not Assessed
Methylation Array			**SRFD**[a]<br>**NNLS**[b]	
WGBS		**cfSort**[c]<br>**Cancer Detector**[d]	**CelFEER**[e]<br>**caHMH**[f]	Sun *et al.*[g]
scRRBS			**caHMH**[f]	
MeDIP-Seq				Shen *et al.**
ELSA-Seq (Targeted Sequencing)				Liang *et al.**
Targeted Sequencing	**TMeF**			

*Shen *et al.* 2018 [ref 42] and Liang *et al.* 2021 [ref 40] demonstrate low quantitative accuracy with a tissue-informed approach utilizing cell line mixtures.
[a]Zhou *et al.* 2022 [ref 43]; [b]Moss *et al.* 2018 [ref 38]; [c]Li *et al.* 2023 [ref 46]; [d]Li *et al.* 2018 [ref 39]; [e]Keukeleire *et al.* 2023 [ref 45]; [f]Guo *et al.* 2017 [ref 41]; [g]Sun *et al.* 2015 [ref 44].

Figure S13. Review of tissue-free, methylation-based ctDNA abundance methods and assays. A 2-dimensional grid depicting assay types (y-axis) and the assessed lower limit of accurate tissue-free quantification (x-axis) for the current presented approach (TMeF) and prior works that utilize methylation patterns to measure ctDNA abundance.
